# Nursing Performance and Mobile Phone Use: Are Nurses Aware of Their Performance Decrements?

**DOI:** 10.2196/humanfactors.4070

**Published:** 2015-04-23

**Authors:** Deborah McBride, Sandra A LeVasseur, Dongmei Li

**Affiliations:** ^1^Samuel Merritt UniversityOakland, CAUnited States; ^2^Hawaii State Center for NursingUniversity of Hawaii at ManoaHonolulu, HIUnited States; ^3^Clinical and Translational Science InstituteUniversity of Rochester School of Medicine and DentistryRochester, NYUnited States

**Keywords:** distraction, mobile phone, cellular phone, Internet, nurses, hospital, non-work related mobile phone use

## Abstract

**Background:**

Prior research has documented the effect of concurrent mobile phone use on medical care. This study examined the extent of hospital registered nurses’ awareness of their mobile-phone-associated performance decrements.

**Objective:**

The objective of this study was to compare self-reported performance with reported observed performance of others with respect to mobile phone use by hospital registered nurses.

**Methods:**

In March 2014, a previously validated survey was emailed to the 10,978 members of the Academy of Medical Surgical Nurses. The responses were analyzed using a two-proportion z test (alpha=.05, two-tailed) to examine whether self-reported and observed rates of error were significantly different. All possible demographic and employment confounders which could potentially contribute to self-reported and observed performance errors were tested for significance.

**Results:**

Of the 950 respondents, 825 (8.68%, 825/950) met the inclusion criteria for analysis. The representativeness of the sample relative to the US nursing workforce was assessed using a two-proportion z test. This indicated that sex and location of primary place of employment (urban/rural) were represented appropriately in the study sample. Respondents in the age groups <40 years old were underrepresented, while age groups >55 years old were overrepresented. Whites, American Indians/Alaskan natives, and Native Hawaiian or Pacific Islanders were underrepresented, while Hispanic and multiple/other ethnicities were overrepresented. It was decided to report the unweighted, rather than the weighted survey data, with the recognition that the results, while valuable, may not be generalizable to the entire US registered nursing workforce. A significant difference was found between registered nurses’ self-reported and observed rates of errors associated with concurrent mobile phone use in following three categories (1) work performance (*z*=−26.6142, *P*<.001, Fisher’s exact test), (2) missing important clinical information (*z*=−13.9882, *P*=.008, Fisher’s exact test), and (3) making a medical error (*z*=−9.6798, *P*<.001, Fisher’s exact test). Respondents reported that personal mobile phone use by nurses at work was a serious distraction; always (13%, 107/825), often (29.6%, 244/825), sometimes (44.6%, 368/825), rarely (8.7%, 72/825), or never (1.2%, 10/825). On balance, 69.5% (573/825) of respondents believed that nurses’ use of personal mobile phones while working had a negative effect on patient care. Since all possible confounders were tested and none were deemed significant, a multivariate analysis was not considered necessary.

**Conclusions:**

Many hospitals are drawing up policies that allow workers to decide how to use their devices at work. This study found that nurses express a disproportionately high confidence in their ability to manage the risk associated with the use of mobile phones and may not be able to accurately assess when it is appropriate to use these devices at work.

## Introduction

Studies examining the effect of mobile phones on work performance have proliferated [1-3], and they have demonstrated that concurrent mobile phone use results in performance decrements, including the impaired ability to focus, difficulty filtering out extraneous information, and the impaired ability to remember important information. In clinical environments, this distraction could cause substandard patient care. Katz-Sidlow et al [4] reported that 19% of residents and 12% of attending physicians acknowledged missing important clinical information because of distractions from mobile phones. In addition, 34% of residents and 20% of attending physicians reported observing another team member miss an important piece of clinical information because they were distracted by their mobile phones during rounds. Smith et al [5] surveyed surgical technicians, of whom 92.7% reported that they had never been distracted by or had their performance negatively affected by their mobile phone, and 98% reported that they had never made an error that could be attributed to their mobile phone use. In contrast, 34.5% reported seeing another surgical technician distracted by their mobile phone during surgery. These results suggest that while many clinicians are aware of the potential dangers of using mobile phones while working, they may not be aware of their own decreased performances. Another study described the underestimation of self-reported medical errors relative to those observed [6]. The researchers identified a self-esteem bias, where respondents have a subconscious tendency to protect themselves from the emotional distress associated with personal deficiencies, resulting in a lack of awareness of medical errors.

The objective of this study was to compare self-reported performance decrements with reports of observed performance decrements related to mobile phone use by registered hospital nurses.

## Methods

We used a survey instrument piloted in 2013 [7]. The survey consisted of four parts, with questions about (1) demographics, (2) the use of personal communication devices, (3) opinions about the effects of personal communication devices on the work of registered nurses, and (4) hospital policies concerning personal communication devices (Multimedia Appendix 1). The questions, which were developed based on a literature review and interviews with hospital nurses, asked respondents to rank the types of activities they engage in on a 5-point Likert scale to determine how frequently they participated in each activity. Psychometric testing of the questionnaire included examining internal consistency reliability and test-retest reliability in a sample of 50 registered nurses. A Spearman rho correlation was used to determine the test-retest reliability. There was strong test-retest reliability between the two tests with a mean agreement for the Likert scale responses of 74% (SD 15, range 43-100%). Accounting for responses within 1 SD range on the Likert scale increased the agreement to 96% (SD 7, range 87-100%). The Cronbach coefficient alpha values examining the internal consistency and reliability in three of the domains were high; utilization (.84), impact (.96), and opinion (.85). A lower agreement was observed in the performance domain (.45). Based on the results of the pilot survey, several questions in the performance domain were rewritten to clarify the underlying concept of work performance.

For the purposes of the study, a personal communication device was defined as a wireless handheld device owned by an individual which can make and receive telephone calls or which provides a connection to the Internet via email, text messaging, videoconferencing, and social networking software. This definition includes cellular phones, mobile phones with app capabilities, and electronic tablet computers, but excludes desktop computers, pagers, or any company-provided device. Of the 825 respondents, 1.3 % (11/825) reported not owning a mobile phone or a comparable mobile communication device. For the purposes of reporting the results of the survey, the term mobile phone is therefore used as a general term to cover the multitude of mobile communication devices currently in use in hospitals.

In March 2014, a recruitment email containing a link to the previously validated 30-question survey was sent to the 10,978 members of the Academy of Medical-Surgical Nurses [8]. The Academy is a specialty nursing organization focused on medical-surgical nursing, with members based across the United States. Membership is open to anyone interested in medical-surgical nursing, including registered nurses, clinical nurse specialists, nurse practitioners, researchers, and administrators.

After excluding all respondents who did not meet the study criteria of having current full-time employment as a registered nurse in a hospital with an average of >5 hours a week of patient contact, the sample was divided into sub-groups for age and ethnicity. The subsets were examined to determine the representativeness of the sample relative to the US nursing workforce [7]. The probability that the proportions of various subgroups in the study sample were representative of the larger population of the US nursing workforce was calculated using a two-population z test to determine whether any weighting was necessary.

The results of the questions about self-reported and observed error rates were analyzed using a two-proportion z test (alpha=.05, two-tailed) to determine whether the differences between self-reported and observed outcomes were significant. All possible confounders which could potentially contribute to self-reported and observed performance errors were tested for significance, including age, race/ethnicity, length of time employed as a registered nurse, location of primary place of employment (inner city, rural, suburban, or urban), job title in primary nursing position, primary nursing role, number of hours per week spent caring for patients in an in-patient setting, US state of employment, type of primary place of employment (for profit, not for profit, or state/local government community hospital), and number of beds at primary place of employment.

## Results

### Overview

A total of 940 (8.56%, 940/10,978) respondents completed the Web-based questionnaire, and 825 (7.25%, 825/10,978) met the inclusion criteria for the study; having current full-time employment as a registered nurse in a hospital with an average of >5 hours a week of patient contact.

### Demographics

Of the study sample, 48 (5.8%, 48/825) were male and 775 (93.9%, 775/825) were female. The age ranges were 20-30 years (9.3%, 77/825), 30-40 years (18.1%, 149/825), 40-50 years (23.9%, 197/825), 50-60 years (39.2%, 323/825), and >60 years (9.3%, 77/825). The results of the two-proportion *z* test indicated that sex and location of primary place of employment (urban/rural) were represented appropriately in the study sample. Respondents in the age groups <40 years old were underrepresented, while age groups >55 years old were overrepresented. Whites, American Indians/Alaskan natives, and Native Hawaiian or Pacific Islanders were underrepresented, while Hispanic and multiple/other ethnicities were overrepresented. Weighting the study sample data for age and race/ethnicity was determined to be undesirable because of the small sample sizes of several age groups and the inherent subjectivity of racial/ethnic groups.

### Effects on Performance of Mobile Phone Use

Three survey questions assessed self-reported and witnessed performance decrements associated with the use of smartphones in the following areas (1) negative performance, (2) medical errors, and (3) missed clinical information. A significantly lower percentage of respondents self-reported mobile phone-related performance decrements (7.4%, 61/825) than reported witnessing mobile phone-related performance decrements in other nurses (70.9%, 584/825, *z*=−26.6142, *P*<.001, Fisher’s exact test) (Table 1).

Significantly fewer respondents self-reported making medical errors (adverse effect of care, including a near-miss or a sentinel event) because of a mobile phone-related distraction (0.8%, 7/825) than reported witnessing such medical errors in other nurses (13.1%, 108/825, *z*=−9.6798, *P*=.008, Fisher’s exact test) (Table 1). Likewise, significantly fewer respondents self-reported missing important clinical information because of mobile phone-related distractions (4%, 33/825) than reported witnessing other nurses missing important clinical information (29.9%, 246/825, *z*=−13.9882, *P*<.001, Fisher’s exact test) (Table 1).

**Table 1:** Questionnaire responses (N=825).

Questions		Response, n (%)	
		Yes	No
**Performance**			
	Has mobile phone use ever negatively affected your performance as a nurse?	61 (7.4)	15 (1.8)
	Have you ever witnessed a nurse colleague’s mobile phone use negatively affect his/her performance?	585 (70.9)	16 (1.9)
	z and Chi-square *P* values^a^	*z*=−26.6142, *P*<.001	
**Medical error**			
	Have you ever made a medical error (an adverse effect of care, including a near miss or a sentinel event) because you were distracted by your mobile phone?	7 (0.8)	34 (4.1)
	Have you ever witnessed a nurse make a medical error (defined as an adverse effect of care, including a near miss or sentinel event) because he/she was distracted by his/her mobile phone?	108 (13.1)	20 (2.4)
	z and Chi-square *P* values	*z*=−9.6798, *P*=.008	
**Missed information**			
	Do you think you have ever missed an important piece of clinical information because you were distracted by your mobile phone?	33 (4)	29 (3.5)
	Have you ever witnessed a nurse miss an important piece of clinical information because he/she was distracted by his/her mobile phone while working?	247 (29.9)	21 (2.5)
	z and Chi-square *P* values	*z*=−13.9882, *P*<.001	

^a^The *z* and Chi-square *P* values are for the difference between self-reported and observed errors in each case. *P* values were calculated using Fisher’s exact test.

### Serious Distraction

Of the 825 respondents, 351 believed that “smartphones can be a serious distraction during work”; always (13.0%, 107/825) or often (29.6%, 244/825). An additional 368 (44.6%, 368/825) believed that mobile phones were a serious distraction sometimes, and 82 thought that they were rarely (8.7%, 72/825) or never (1.2%, 10/825) a distraction. As well, a fraction, (2.9%, 24/825) did not answer the question.

### Balance of Negative or Positive Effect on Patient Care

When asked, “On balance, do you think the use of personal smartphones by nurses while working has a more positive or negative effect on patient care?”, the majority, 69.5% (573/825), said it has a more negative effect. Only 27.5% (227/825) said that the effect was more positive, and 25 respondents (3.0%, 25/825) did not answer the question.

### Confounding Variables

Since all possible confounders were tested and none were significant, a multivariate analysis was not considered necessary. The proportion test was conducted under the assumption of normal approximation to the binomial distribution. The conditions for the normal approximation from the binomial distribution were satisfied. More advanced data analysis was not conducted because no confounding variables were significant.

## Discussion

### Principal Findings

An overwhelming majority of respondents answered that their work performance had never been negatively affected by their mobile phone use, with only 61 (7.4%, 61/825) reporting negative effects on performance, 7 (0.8%, 7/825) reporting that they had made a clinical error, and 33 (4%, 33/825) saying that they had missed an important piece of clinical information. However, far more said that they had witnessed a nurse colleague’s work performance negatively affected due to the use of a mobile phone, with significant differences across all three categories. For example, 585 respondents (70.9%, 585/825) reported that they had seen a colleague’s work performance negatively affected. More than 100 reported seeing someone else make a clinical error, and over one quarter claimed that they had seen a colleague miss a significant piece of clinical information.

### Comparison With Prior Work

These results are consistent with findings from previous studies, including Katz-Sidlow et al’s work on residents and attending physicians [4], and Smith et al’s study of surgical technicians [5]. Both reports found significant discrepancies between self-reported and observed effects of mobile phone use on performance among the groups studied. There are several possible reasons for these discrepancies, including multiple observations of a smaller number of errors. However, this is unlikely in this study given the relatively small number of respondents coming from a wide geographical area, and the large differences between self-reported and observed outcomes. It is more likely that nurses may not be aware of their own mistakes while using their mobile phones or may not believe that they are distracted by them.

This conclusion is supported by work in other fields. For example, Lesch and Hancock [9] carried out a study to determine whether drivers were aware of their reduced driving ability while operating a mobile phone. They found that drivers were oblivious to their reduced driving ability caused by concurrent mobile phone use. Strayer et al [10] found that drivers described other drivers using their cell phones as driving poorly but reported that their own driving during mobile phone use remained normal, even when the results of driving performance tests showed otherwise. These results support our findings and suggest that there is a disconnect between self-reported and observed performance among respondents. Although respondents self-report low levels of performance decrements, the significantly higher level of reported witnessed performance decrements should be a cause for concern because it raises the possibility of substantial patient safety issues.

A recent study found that medical students believe that using mobile phones to support clinical work enables them to work more efficiently [11]. However, there were serious concerns raised in that study about the security of patient data transmitted via unsecured mobile phones, not least that current use may not be consistent with regulatory requirements. Similar to our results, this suggests that staff may be unable to accurately assess the risks of mobile phone use.

### Limitations

This study has some limitations. First, the sample subgroups are not representative of the wider nursing population. Consideration was given to weighting the study sample data for race/ethnicity and age, but several points argued against it, such as the small sample sizes within several age groups and the inherent subjectivity of racial/ethnic groups. While the response rate was low relative to other online surveys, this may have been the result of the perceived sensitive nature of the subject, with respondents preferring not to admit that they had used their personal communication devices at work for non-work related activities. Holbrook et al [12] assessed whether lower response rates are associated with less unweighted demographic representativeness of a sample. By examining the results of 81 national surveys with response rates varying from 5%-54%, they found that surveys with much lower response rates were only minimally less accurate. As such, it was decided not to weight the current survey data, but to report the unweighted survey results with the recognition that the results, while valuable, may not be generalizable to the entire US registered nursing workforce.

Second, there may have been some selection bias. The usable response rate was quite low (7.25%, 60/825) as nurses may have been reluctant to participate because of the sensitive nature of the topic. This may have been a particular problem for any nurses who felt that their own performance had suffered as a result of personal mobile phone use and as such, the study overestimated the differences between the two groups. However, because these differences were quite large, this may not have affected the derived conclusions.

Finally, the results were based on respondents’ recall and may not reflect current usage patterns. Real-time observations of mobile phone usage would provide a more accurate description of current usage patterns.

### Conclusions

In response to concerns about mobile phone use, many hospitals are drawing up policies outlining the appropriate use of these devices at work. One approach is to allow workers to decide how and when to use their devices at work. This presumes, however, that workers can accurately assess the risks associated with the use of their mobiles phones in a workplace environment and can appropriately modify their behavior. The results of this study indicate that registered nurses may express a disproportionately high confidence in their ability to manage the risk associated with the use of mobile phones at work relative to other registered nurses’ performance, and may not be able to accurately assess when it is appropriate to use them and to modify their behavior accordingly.

## Multimedia Appendix 1

Survey instrument used in the study.
